# A socio-ecological perspective of access to and acceptability of HIV/AIDS treatment and care services: a qualitative case study research

**DOI:** 10.1186/s12889-016-2830-6

**Published:** 2016-02-16

**Authors:** Bereket Yakob, Busisiwe Purity Ncama

**Affiliations:** School of Nursing & Public Health, Howard College, University of KwaZulu-Natal, King George V Ave, Durban, 4041 South Africa; Health Economics and HIV/AIDS Research Division (HEARD), University of KwaZulu-Natal, Durban, South Africa

**Keywords:** Access, Acceptability, HIV/AIDS, Responsiveness, Socio-ecological perspective, Wolaita Zone, Ethiopia

## Abstract

**Background:**

Access to healthcare is an essential element of health development and a fundamental human right. While access to and acceptability of healthcare are complex concepts that interact with different socio-ecological factors (individual, community, institutional and policy), it is not known how these factors affect HIV care. This study investigated the impact of socio-ecological factors on access to and acceptability of HIV/AIDS treatment and care services (HATCS) in Wolaita Zone of Ethiopia.

**Method:**

Qualitative case study research was conducted in six *woredas* (districts). Focus group discussions (FGDs) were conducted with 68 participants in 11 groups (six with people using antiretroviral therapy (ART) and five with general community members). Key informant interviews (KIIs) were conducted with 28 people involved in HIV care, support services and health administration at different levels. Individual in-depth interviews (IDIs) were conducted with eight traditional healers and seven defaulters from (ART). NVIVO 10 was used to assist qualitative content data analysis.

**Results:**

A total of 111 people participated in the study, of which 51 (45.9 %) were male and 60 (54.1 %) were female, while 58 (53.3 %) and 53 (47.7 %) were urban and rural residents, respectively. The factors that affect access to and acceptability of HATCS were categorized in four socio-ecological units of analysis: client-based factors (awareness, experiences, expectations, income, employment, family, HIV disclosure and food availability); community-based factors (care and support, stigma and discrimination and traditional healing); health facility-based factors (interactions with care providers, availability of care, quality of care, distance, affordability, logistics availability, follow up and service administration); and policy and standards (healthcare financing, service standards, implementation manuals and policy documents).

**Conclusions:**

A socio-ecological perspective provides a useful framework to investigate the interplay among multilevel and interactive factors that impact on access to and acceptability of HATCS such as clients, community, institution and policy. Planners, resource allocators and implementers could consider these factors during planning, implementation and evaluation of HATCS. Further study is required to confirm the findings.

**Electronic supplementary material:**

The online version of this article (doi:10.1186/s12889-016-2830-6) contains supplementary material, which is available to authorized users.

## Background

Access to healthcare is an essential element of health development and a fundamental human right k. However, due to the different developmental status and strategies used, access to healthcare varies across and within nations. Infectious diseases with wider scope and impact, specifically HIV/AIDS, competing health priorities, the demand for advanced care and strained resources deter efforts to ensure universal access to healthcare [1]. Increased stewardship and solidarity from governments and the global community has enabled substantial improvement in access to HIV/AIDS treatment and care services (HATCS) in the past decade [2]; nearly 15 million (41 %) people living with HIV (PLHIV) were on antiretroviral therapy (ART) in 2015 [3]. However, despite this progress, about 49 % of PLHIV did not know their HIV status in 2014, thus limiting entry to HATCS [3, 4]. According to the HIV/AIDS fact sheet update of March 2015, 70 % of all PLHIV lived in sub-Saharan Africa and 66 % of all deaths related to HIV/AIDS occurred in this region [3]. Ethiopia has a high burden of HIV with over 800 000 PLHIV of which only 344 344 (43 %) accessed ART in 2014 [5].

Access to HATCS in developing countries is further impacted by stigma, misconceptions, and the poor quality of care and unacceptability of services [6–8]. Acceptability of healthcare is a concept that is embodied in clients and is thus affected by perceptions of the accessibility of the healthcare, ‘health system responsiveness,’ and clients’ psychological status, experiences and expectations [9, 10]. Each service obtained from health facilities that constituted clients’ experience of healthcare, is evaluated in terms of ‘the ways and environment in which care is provided’ or *responsiveness* [11] and financial fairness [12]. Based on the health care climate experienced, quality of care is perceived; this ultimately impacts client satisfaction, adherence to prescriptions and treatment outcomes [12]. However, acceptability of care has not been a priority in the evaluation of health systems’ performance and/ or health services research, especially in relation to HATCS.

With the new World Health Organization (WHO) guideline on when to start ART, that is to provide ART for all PLHIV and pre-exposure prophylaxis to all people with increased risk of acquiring HIV [13], the logistics, management and services challenges will mount, especially for resource-limited countries [14]. Against this backdrop, governments need to design doable and effective strategies to ensure that all PLHIV have access to HATCS. Thus, evidence is required that will enable resources to be committed to acceptable health interventions that have high health impact. The above discussion shows that multiple factors interplay and impact access to and acceptability of healthcare. This study, therefore, used the socio-ecological model to investigate multilevel and interactive factors such as individual, community, institutional and policy factors that impacted access and acceptability of HATCS in Wolaita Zone of Ethiopia. It explored the different aspects of these factors that acted either as barriers or enhancers of access to and acceptability of HATCS.

## Methods

### Study area

The study was conducted in Wolaita Zone of the Southern Nations, Nationalities and Peoples Region (SNNPR) of Ethiopia which is located 330 km south west of Addis Ababa (the capital city). Based on the national population projection for 2014, 1 866 400 people lived in the zone, of which males and females accounted for 49.3 % and 50.7 %, respectively. According to the 2007 Ethiopian Census, Christians accounted for 98 % of the population while the rest were affiliated to Islam and other traditional beliefs. Most of the population was ethnic Wolaita and 96.8 % spoke Wolaita Donna. The zone was administratively divided into 15 *woredas* (equivalent of districts), including three towns and 12 rural *woreda* administrations. The *woredas* were further segmented into 346 *kebeles*, the smallest administrative unit equivalent to or an aggregate of villages. In 2014, the potential health coverage of the zone was reported to be over 95 %, and three hospitals (one public and two private), 63 health centers (all public) and 333 health posts were in service. There were 16 795 people living with HIV (PLHIV), estimated using the pooled SNNPR’s HIV prevalence rate of 0.9 %, of which 3 038 were ‘currently on ART’. The zonal health sector performance report for 2014 acknowledged challenges to the utilization of HIV treatment and care services such as low ART coverage, lost to follow up and staffing problems (Yakob B & Ncama BP, unpublished observation). This further motivated this study.

### Study design

A qualitative case study research was conducted with a focus on exploring and explaining the factors affecting access to and acceptability of HATCS. Case study research enables the use of multiple sources of information to build and test theories, and to better understand social phenomena [15]. The ‘case’ of the study was Wolaita Zone that was bounded a) temporally - from November 2014 to April 2015 b) programmatically - HIV treatment and care services and c) conceptually - factors related to and affecting access to and acceptability of HATCS. In order to enable systematic inquiry, embedded units of analysis were identified in recognition of the complexity of the phenomena after reviewing the widely used general socio-ecological theories such as *The Ecology of Human Development* [16], *Ecological Model for Health Promotion* [17] and the *Social-ecology Framework* [18]. These models provided a general framework of systems and interactive levels such as intrapersonal, interpersonal, institutional, community and policy that assisted in explaining the factors related to access to and acceptability of HATCS (Table 1).

**Table 1:** Theoretical frameworks, unit of analysis and propositions of the study

Ecological model for health promotion or Socio-ecological model (McLeroy et al (1988))	Ecology of Human Development (Bronfenbrenner (1979))	Social-ecological Model (Dahlberg and Krug (2002))	Unit of analysis adapted and proposed	Propositions of the study
Intrapersonal (biology, knowledge, self-concept, attitudes, etc.)	Microsystem (individuals’ direct interaction with objects and related people)	Individual (biological and socio-demographic factors)	Client-based	Client-based factors influence the acceptability of HATCS
Interpersonal (interactions with other people and groups)	Mesosystem (interaction with groups and networks of people, i.e., peers and churches)	Relationships (interaction with one or more people)		
Institutional (interaction with social institutions and structures)	Exosystem (a factor indirectly influencing a person due to its influence on a related person)		Community	Community-based factors influence access to and acceptability of HATCS
Community (interactions between organizations)		Community (schools, workplaces, neighborhoods, and other organizations)	Institutional	Institutional factors influence access to and acceptability of HATCS
Policy (laws, policies, standards, directives, implementation manuals, etc.)	Macrosystem (policy and societal factors i.e., culture)	Societal (policies, national economic performance, etc.)	Policy and standards	Policies and standards influence access to and acceptability of HATCS

After reviewing a number of theories on access to healthcare [10, 19–22] and evaluating these against the socio-ecological frameworks discussed above, four embedded units of analysis were identified: client-based, community-based, institution-based and policy. Corresponding propositions were stated for each unit of analysis (Table 1) to guide the enquiry and analytic processes [15]. The study sought to determine what constituted each embedded unit of analysis, how they interacted and what influence they had on access to and acceptability of HATCS. For clarity, the following definitions of access to and acceptability of healthcare were used.

*Access is the extent to which the health care system fits, inhibits or initiates individuals’ willingness and ability to enter, receive and benefit from the outcomes of, and to gain satisfaction from health services. It is the process of knowing about, seeking, entering, passing through and obtaining satisfaction from the care and benefiting from the outcomes of the health service, and is not restricted to merely consulting the health care provider and/ or getting prescriptions* (Yakob B & Ncama BP, unpublished observation). Acceptability is the degree to which the services delivered by the healthcare organizations satisfy the individual’s expectations, personal preferences and wishes.

### Sample size, sampling procedures and data collection

A mix of purposive sampling methods were used based on the availability of potential participants and need. The sampling procedures selected and discussed below were informed by Yin [15] and Bryman [23]. For instance, *criterion sampling* was conducted to select six *woredas* of which three were urban and three were rural by virtue of manageability, affordability and availability of HATCS. The three town administrations in the zone and two rural *woredas* had comprehensive HATCS (ART, pre-ART and other HIV/AIDS care services). However, one rural *woreda* was the most remote and did not have comprehensive HATCS (i.e., lacked ART care), thus ensuring *maximum variation sampling*. This was also achieved by stratifying the sampling units on residence (urban/rural), service availability (*woredas* with and without ART services) and role in HIV care (clients, care providers and health administrators). Potential participants with different attributes such as with experiences and awareness as PLHIV, using HATCS, stopped ART care, healthcare providers, traditional healers, health administrators and officers, coordinators of PLHIV associations, community volunteers and workers, and community members were invited to participate in the study. For FDGs and IDIs, the *theoretical sampling* method was used and sampling continued until *theoretical saturation* was achieved.

FGDs were conducted with 68 participants in 11 groups (six with people using ART and five with general community members). KIIs were conducted with 28 persons involved in HIV care, support services and health administration at different levels and IDIs were conducted with eight traditional healers (three prayer service providers and five herbalists) and seven defaulters from ART. Details of the sampling procedures and inquiry types are presented in Table 2. In addition, observations of seven health facilities (one hospital and six health centers) were conducted with checklists.

**Table 2:** Sampling and sample size

Inquiry type	Gender	# participants (Rural woredas with ART)	# participants (Rural woreda without ART)	# participants (Urban woredas with ART)	Total
FGDs with PLHIV	M	5	0	12	17
(3 M & 3 F groups)	F	7	0	12	19
FGDs with community	M	7	0	6	13
(2 M & 3 F groups)	F	7	6	6	19
IDIs with defaulters	M	2	0	1	3
(stopped ART)	F	1	0	3	4
IDIs with traditional	M	1	1	1	3
healers	F	1	1	3	5
KIIs with health	M	2	3	5	10
administrators	F	0	0	0	0
KIIs with care	M	0	1	2	3
providers	F	2	0	2	4
KIIs with community	M	0	0	0	0
level workers	F	4	1	4	9
KIIs with APLHIV	M	1	0	1	2
heads	F	0	0	0	0
Total	M	18	5	28	51
	F	22	8	30	60
	Total	40	13	58	111

*FGDs* focus group discussions, *IDIs* individual in-depth interviews, *KIIs* key informant interviews, *APLHIV* association of people infected with HIV, *ART* antiretroviral therapy, *M* male, *F* female

### Data collection tools

Five related but different semi-structured interview and open-ended FGD guides were developed by the investigators and were scrutinized by experts (a panel of PhD students in public health and professors) before data collection. Interview guides were followed by probes and/ or follow up questions as needed. The guiding questions focused on views about HATCS, experiences with health facilities, the challenges of using HATCS, what makes for good care, the role of traditional healing, what traditional healing was available and what improvements should be made to improve access to and acceptability of HATCS. Details on the IDIs and FGDs guides can be found in Additional file 1. A health facility observation checklist was developed after reviewing the literature and mainly focused on health service flow and amenities of care (cleanliness, walkways, toilets, linen and availability of electricity and water).

### Data collection

Data were collected by the PI, assisted by a research assistant (RA) who was a fluent speaker of the local languages (Wolaita Donna and Amharic) with a Master’s of Public Health and experience in qualitative research. The RA was trained for two days and was involved in preparation for data collection (FGDs and IDIs) and field note taking. Participant recruitment was assisted by health care providers and the PLHIV associations. They identified and contacted potential participants with the desired attributes and linked with the PI once they had obtained agreement to participate. KIIs and IDIs were conducted in either their offices, homes or other places of their choice where confidentiality and privacy were maintained. All interviews but five were recorded after obtaining the participants’ permission. When the participants did not want the audios of the interview recorded, notes were taken, the information was retained and analyzed along with other data. FGDs were conducted in *kebele* meeting halls, health posts or PLHIV associations’ offices. Written consent was obtained from all participants after orientation.

### Data analysis

Qualitative content analysis was performed with the steps being informed by Miles, Huberman and SaldañA [24] and Schreier [25] recommendations. All data were transcribed verbatim and translated into English by the PI. The transcripts were read and reread to gain familiarity with the content and were checked against the recordings to confirm or correct any errors. The data were later anonymized and impressions from observations and field notes were inserted. NVIVO 10 (QSR International Pty Ltd.) was used to assist with data organization, condensation and analysis. The ‘first cycle coding’ was mainly data-driven (in vivo), while the ‘second cycle’ (pattern matching) coding emphasized bringing together those codes with similar or related concepts to create small categories. As the analysis was theory-driven, the relationships between the second level codes were examined and grouped in such a way that provided analytic meaning that was later referred to higher level coding or category. In this later stage, categories were compared against the embedded units of analysis and revised. This process was supported by the *analytic memoing* during the coding phases. The categories were further examined to determine whether they fitted the concepts, propositions and theories in the socio-ecological frameworks [16–18, 26] and literature review [10, 19–22]. Throughout data collection, field notes were taken and preliminary methodological and analytic ‘memoing’ were conducted. In addition, the investigators reevaluated, revised and reconfigured the codes iteratively until consensus was reached.

### Trustworthiness

Data were collected from multiple sources, i.e., from people with different experiences, health conditions and responsibilities that permitted *maximum variability* and/ or *theoretical saturation*. In addition, a narrative *thick description* was conducted that enhanced *rigor* and *transferability* of the findings. Preliminary findings were presented, discussed and validated with experts (professors in the field) and people involved in HIV care in the study area [27]. A *case study protocol* that included the purpose and design of the study, data collection and data analysis procedures and reporting strategies [15] was developed in advance and maintained throughout the study. In addition, the data collection instruments were developed after triangulating theories, literature review and expert panel discussion to ensure *dependability* of the study. All data collected from the field work were maintained and were frequently consulted, discussed and agreed between the investigators during the analysis and interpretation phase that ensured *confirmability* of the findings. The study instruments were translated into local languages and the inquiry was conducted in the language of the participant’s choice to reduce bias and errors. Furthermore, the PI and RA, lived in the study area, are fluent in both local languages and knew the culture, and this reduced the possibility of misunderstanding during the study but ensured *credibility* of the findings [27, 28]. All field notes were saved and assisted in reflexivity and iterative data analysis.

### Ethical considerations

The study was approved by the IRB of Wolaita Soddo University (Ethiopia) and the Bio-medical Research Ethics Committee (BREC) of the University of KwaZulu-Natal (South Africa). FGD participants were reimbursed 100ETB (~USD$5) for the time spent in the discussion and for transport expenses. On obtaining their permission, the study participants who had stopped ART were counseled and linked with the care providers in their respective areas and restarted HIV treatment. All audio recordings were transferred to a password protected computer and anonymized. All transcripts were anonymized ahead of data analysis.

## Results

The findings of the study are presented below in terms of the participants’ characteristics and the four embedded units of analysis: client factors, community factors, healthcare factors and policy and standards.

### Participants’ characteristics

A total of 111 people participated in the study, of which 51 (45.9 %) were male and 60 (54.1 %) were female, while 58 (53.3 %) and 53 (47.7 %) were urban and rural residents, respectively. Overall, 23 (20.7 %), 53 (47.6 %), 13 (11.7 %) and 22 (19.8 %) of the participants were in the age group <30 years, 30-39 years, 40-49 years and >49 years, respectively. The mean age was 36.3 years, with 35.8 years for FGDs, 33.9 years for KIIs and 42.7 years for IDIs. In addition, 45 (40.5 %) of participants were HIV positive while 66 (59.5 %) had unknown HIV status. Marital status was available for FGD and II participants of whom 62 (74.8 %) were married, five (6.0 %) were single, eight (9.6 %) were divorced and eight (9.6 %) were widowed. All the participants were affiliated to different Christian denominations.

### Client factors

#### Awareness and experiences

Awareness of HIV/AIDS treatment increased among PLHIV over the years as a result of observing someone that benefitted from HIV treatment and/or witnessing the death of those who refused to start or failed to adhere to HIV treatment. In addition to knowing one had HIV, these experiences influenced the client’s outlook on life and decisions on when to start and adherence to HIV treatment. PLHIV associated late diagnosis and delayed HIV treatments with poor outcomes even if put on appropriate treatment. Drug side effects were misconceived by some PLHIV and caused them to stop treatment.

*Many people died of HIV/AIDS because they did not use the treatment or follow prescriptions. … I have seen people using HIV drugs benefited. … In my case, the disease severed my health because it was diagnosed very late.* (FGD 5, 35 year old woman, using ART)

*Currently, the drug they are giving … I tell you! … It’s very strong and burns your stomach … It makes you lose all your energy. … That I stopped the drugs.* (II 6, 31 year old woman, stopped ART)

New users of HIV treatment often feared the side effects of the drugs and were uncertain about the benefits. This tended to be resolved over time. They reported that the initial experiences with care providers were crucial to clear any confusion and misunderstanding and to respond to clients’ concerns in order to encourage them to adhere to treatment and start living positively.

*There are many bad things said about starting HIV drugs … like it can kill the person quickly or worsen the health condition. So, they [PLHIV] come with many confusions, concerns and questions. Health care providers must understand these issues and address appropriately. Without good counseling and care in their first visit, it will affect adherence and positive living. Some people even might quit the drug shortly.* (FGD 3, 35 year old woman using ART)

Some PLHIV believed that being HIV positive meant that the virus had depleted their blood to the extent that drawing even a small blood sample for laboratory investigations adversely affected their health. In addition, PLHIV frequently associated HIV drugs with “feeling hungry,” “stomach burning” (heartburn, gastritis or ulcers), “body weakness” and/ or “diarrhea”. Due to these beliefs, some demanded adequate food as a basic requirement to stay in HIV treatment.

*I refused to give blood for testing [CD4 cell count] … because I did not want to lose my already depleted blood.* (II 8, 35 year old man, stopped ART)

*When I took the drugs in empty stomach, it burned me [gastritis, heart burn or ulcer] that followed with diarrhea and body weakness. So, I stopped it [HIV drugs].* (II 7, 38 year old woman stopped ART)

#### Resources (Food, income and employment)

Employment opportunities and the ability to buy food were associated with the acceptability of HIV care as participants believed that HIV care should be comprehensive and should include food. Both the community and PLHIV believed that HIV positive people should eat “good foods” such as meat, eggs, butter, milk, etc. Unemployment resulted in low income, hindering access to such foods and increasing worries about health and the future. The inability to provide food, clothing and school materials for their children and pay rent caused some PLHIV to abandon HIV treatment. Some PLHIV who stopped ART said that they were depressed and not sure if they would restart ART.

*We can’t take the drugs without food. … HIV treatment should be complemented with food … good foods like meat, egg, milk and so on. Without this, HIV care is unacceptable for me.* (FGD 2, 40 year old man, stopped ART)

*I’m unemployed. I have children to take care of. I have to provide them with food and cloth, and pay my house rental. If I don’t pay on time, they [house owners] will expel me. I have too much to bear at the moment, it is very depressive. I just feel my days are over. So, I stopped ART.* (II 6, 31 year old woman, stopped ART)

#### HIV status disclosure

PLHIV who did not disclose their HIV status to anybody faced challenges in adhering to HIV treatment. For instance, efforts to hide their HIV status from family members (for example, their mother) led to treatment being stopped. The participants also noted that some PLHIV had not disclosed their status to their spouses and that many women and children were thus not accessing HIV care. Care providers and PLHIV reported that many women and children died without knowing they had HIV and only the lucky ones discovered it years after their husbands’ death. Stigma and discrimination and the desire to preserve relationships were the reported causes of failure or unwillingness to disclose one’s HIV status and/or non-use of HIV care.

*I stopped HIV drugs due to the inconvenience I am facing, my mother is sick and I am staying with her to take care of her. I haven’t disclosed my HIV status. I have stopped taking HIV drugs because I don’t want her [my mother] to discover my HIV status.* (II 12, 27 year old woman, stopped ART)

*There are some men who keep their status hidden from their wives although they are using HIV drugs. … As a result, many women and children died without knowing they had HIV. They had no chance of using HIV treatment. Some lucky women got tested years after their husbands’ death and started HIV treatment*. (FGD 9, 25 year old woman, general community member

### Community factors

#### Community acceptance and responses

Both community members and PLHIV believed that increased awareness of HIV/AIDS and the availability of treatment contributed to reduced stigma and discrimination. In effect, PLHIV were integrated in all social activities and were also provided with care and support when they needed it. However, some stigma still remained such as refusing to let houses to those that are HIV positive, stigmatizing the children of PLHIV and insults and name calling that caused people to stop treatment.

*Now, everybody knows about HIV … We have seen the benefits of HIV drugs and their impacts. … We are supporting them whenever they needed financial or food assistance.* (FGD10, 31 year old man, general community member)

*Stigma and discrimination are not over yet. There are some people who say ‘You are HIV,’ ‘an HIV man or woman,’ ‘your days are numbered,’ ‘you are dying,’ ‘you will die soon,’ ‘you are spoiled’ and many other things. Some people stigmatize our children. Due to this, some people stopped HIV treatment.* (KII15, head of *woreda* health office)

It was reported that the care and support (financial, food or material) available to PLHIV were feeble, inadequate and unorganized. Some churches only provided food and/or money during some national holidays. In some *woredas*, the health offices supported PLHIV in the form of seed money to establish small enterprises although such support was said to be insufficient with limited reach. PLHIV associations provided food, money and emotional support to their members during times of illness and a shortage of food. The associations made home visits and offered counseling services, and also assisted health facilities to identify and trace those lost to follow up.

*We are not getting support from the government as well as from the community. The churches give us some money and food during national holidays. We need … seed money for small enterprises and food support to survive.* (FGD3, 31 year old woman, using ART)

#### Traditional healing

In the community, there were traditional healing services in the form of prayers, holy water and herbal medicine. Many PLHIV were using these services with varying beliefs and trust put in them, and the impact on HIV treatment and care varied accordingly. Some PLHIV used traditional healing simultaneously with ART. Others used traditional healing with inconsistent use of ART.

*For instance, I pray and fast and also simultaneously use ART. I take it correctly.* (FGD3, 28 year old woman, using ART)

*As a religious practice, I use holy water every day. … Sometimes I forgot to take the drugs after the holy water. … I didn’t take the drugs as prescribed but I used arbitrarily when it was convenient to me. Later on, I stopped HIV treatment due to lack of hope in life.* (II11, 29 year old man, stopped ART)

Finally, some PLHIV fully relied on traditional healing services and stopped ART. In the IDIs with herbalists, they said they did not provide any treatment for HIV or they did not have a cure. However, PLHIV said they were asked by herbalists to pay money for a cure for HIV. Some said that they knew of people who had stopped HIV treatment and/ or died due to herbal medicine. Claims of being healed were associated with traditional healing and were the cause of stopping ART.

*There was a woman who died after stopping HIV treatment. She was told healed by a prayer in a church service and she stopped ART. There are also other people who stop HIV treatment due to herbal medicine.* (FGD5, 35 year old woman, using ART)

*I don’t provide treatment for HIV. When I provide care, I don’t know who is HIV positive or not, or they don’t disclose their HIV status to me. … When I suspect they have HIV, I tell them to get tested for it.* (II3, 35 year old woman, herbalist)

*There was an herbalist who asked me to pay 3000ETB [~US$150] to get HIV cure. I didn’t trust him because I knew there was no cure for HIV.* (FGD3, 35 year old woman, using ART)

### Healthcare factors

#### Interaction with care providers

Clients’ experiences of their interactions with care providers affected their perceptions of the quality of care that in turn had implications for acceptability of care. The reported acceptable interactions were a welcoming reception, follow up on how they were doing, answering questions, attending and responding to their concerns and empathy. The reported “unacceptable” or “disappointing” interactions were when care providers disrespected patients, did not answer questions and had an unwelcoming look on their faces.

*The health center as well as care providers are very good. They ask us how we are doing and if we have any concerns. They answer our questions. … We are happy with the care and access to care. Usually, our expectations are met.* (FGD1, 30 year old woman, using ART)

*Unfortunately, the care provider was so disrespectful. She didn’t answer my questions appropriately and she shouted at me. Very frustrating! …That is unacceptable! So, I went back home to come again when the main care provider returned.* (FGD2, 40 year old man, using ART)

Some PLHIV had unpleasant experiences with “new care providers” assigned to HIV care units or “filling in” when the normal care provider was absent. Explaining their medical history and their concerns to somebody who would not be there the following month was a “disappointing experience.” In addition, PLHIV were concerned about new workers changing treatment plans without ‘good’ reason. Some PLHIV who had “unpleasant interaction” with care providers stopped ART. In addition, a “lack of empathy” from care providers impacted on satisfaction with services and adherence to treatment.

*Every time we visit the unit, we find a new care providers. …Every time we have to explain everything from the beginning to a new care provider which is not acceptable. …. Besides, they quickly change the drugs that affects our health.* (FGD6, 50 year old man using ART)

*The health care at the health center used to be good before those good care providers had left. The ones providing care [ART] at the health center are disrespectful, very arrogant and not caring. I was so disappointed with their behavior and stopped HIV treatment.* (II10, 55 year old woman, stopped ART)

When PLHIV felt that the care providers stigmatized and/ or discriminated against them, for instance skipped physical examinations or made unnecessary referrals to other health facilities, they became “disturbed” and “hopeless”.

*They [care providers] do not want to touch us or perform physical examinations. We think they are stigmatizing us. …They order lab examination and prescribe based on the lab findings. It’s like a machine operating on us.* (FGD5, 55 year old woman, using ART)

#### Quality of care

Some PLHIV who were accessing HIV care believed that the health care providers followed standard procedures and that the care was “good” and “acceptable.” They believed that they were obtaining appropriate treatment for their health conditions. However, others perceived “poor quality of care” due to a lack of access to a dedicated physician and/ or specialist medical attendant.

*We don’t have physicians working in the unit [HIV unit]. Staffs are assigned on rotational basis which is not a good practice for PLHIV. We also don’t have specialists to consult when we have severe medical problems.* (FGD6, 42 year old man, using ART)

On the other hand, health care providers believed they provided good or quality care because they followed the national and WHO HIV/AIDS service delivery standards. However, they experienced malpractices such as wrong medicine dispensed by new staff. PLHIV also reported that in some cases, HIV care was provided by staff without on-the-job training on HIV treatment. Poor management of the side-effects of HIV drugs and the lack of free treatment for opportunistic infections (OIs) also cased disappointment and unacceptability of HIV care. In addition, a shortage of resources such as laboratories and examination tools and drugs challenged the provision of standard services.

*In the health center, I witnessed a wrong drug [not prescribed] mistakenly dispensed by a new pharmacy technician which resulted in undesired side effects on clients.* (KII7, 25 year old, care provider)

*There is a care provider who is not trained on ART/HIV treatment providing care. He sometimes messes up the treatment regimens. … So, the quality of care is not good.* (KII17, 30 year old, adherence supporter)

*I had itching and eruptions on my skin and I consulted the care provider [at the hospital] and he replied ‘Except for the ART drugs, you must pay for all other medications.’ … They don’t care when we have side effects. … The service is incomplete and is not acceptable by any means. I stopped ART.* (II7, 38 year old female, stopped ART)

#### Follow up

HIV care providers, adherence support workers and case managers said that they followed up on clients via telephone and home visits and during health facility visits. For PLHIV, follow up was another area associated with the acceptability of HIV care in health facilities. Follow up was perceived as “good” when PLHIV were asked how they were doing and adherence to treatment. A lack of follow up resulted in “disappointing” experiences with services. Home visits by adherence supporters were welcomed by PLHIV although this was often not available and was not practiced as promised. PLHIV appreciated the efforts of PLHIV associations to trace lost to follow up and to assist them in dealing with their life problems.

*So far, the health services have been good. … If we don’t appear on the appointment dates, they make calls and follow up. They are doing a good job.* (FGD3, 30 year woman, using ART)

*Previously, they used to ask how we were taking the drugs or if we had encountered problems with the drugs. Now, they prescribe drugs and that’s all! They don’t make follow ups.* (FGD5, 36 year old woman, using ART)

#### Laboratory services and logistics

In addition to concerns about providing “large” blood sample for CD4 cell counts, PLHIV expressed their disappointment with delays in laboratory results because they were eager to know how they were doing. Logistics and supplies challenges were reported by PLHIV, care providers and health administrators. For instance, some health facilities had run short of HIV drugs at least once in the 12 months prior to the time of the study. Drug shortages were managed differently by care providers and health facilities; some health centers borrowed one or more drug items from other facilities to cover the deficit while others did not do so.

*They take blood sample for CD4 count … But, they are not telling us the results, they take ages to do so. That is not acceptable. … For us, knowing our CD4 count is very important.* (FGD2, 25 year old man, using ART)

*Sometimes, we fall short of ART drugs. … When it happens, we borrow from other health facilities in order that our clients do not interrupt the drugs.* (KII6, 26 year old female, care provider)

In the latter health facilities, PLHIV were forced to interrupt treatment for a couple of weeks. Pediatric ART drugs were still not available or were insufficient, challenging parents and health facilities. While resources to treat OIs were usually available in health centers, hospitals faced challenges due to insufficient supplies and large volumes of clients; this was the main cause of dissatisfaction with HATCS in hospitals. In both health centers and hospitals, laboratory services were affected by a shortage of reagents and chemicals that caused delays in results.

*I visited the health center for drug refill [ART] but they said they didn’t have the drugs. After two three attempts, I was disappointed and stopped looking for it.* (II11, 28 year old man, heal of health office)

*We experience delays in logistics supply for HIV treatment such as for the treatment of opportunistic infections. The problem is huge in hospitals because they serve many clients. This is not a case in health centers.* (KII19, 37 year old man, HIV/AIDS program coordinator)

#### Amenities of care

Some health facilities had space problems and poor room arrangements which failed to maintain privacy and confidentiality. For instance in one health center, services providers with different capacities such as receptionist, counselors and HIV care provider all sat in the same narrow room.

*When you visit the health center, you have many things you want to consult with the care provider. In the presence of three or four people in a room, it’s difficult to tell our concerns and secrets to the care provider.* (FGD6, 46 years old man, using ART)

Three health centers lacked space to store logistics for HATCS. One health center had poor infrastructure (blocks) which were difficult to keep clean. Some health facilities also lacked running water in HIV care units as well as an adequate number of toilets. In almost all health facilities, toilets lacked cleanliness and running water and were sometimes out of order or closed. A shortage of chairs was observed in the hospital waiting area and PLHIV queued for long time, especially in the morning sessions. Except for the hospital, all the health facilities lacked access for people with disabilities.

### Operational hours, staffing and administration

Operational hours were cited as an important component that affected the acceptability of care and satisfaction with services. During normal working hours, i.e., 8:30 am – 5:30 pm on weekdays, PLHIV faced few challenges in obtaining care except when care providers were absent. However, many of the health centers did not provide ART or free treatment of OIs at night and on weekends, limiting access to care. Health care providers at some health centers acknowledged the unavailability of after hours’ care that challenged PLHIV’s access to emergency care and clients described this as “disappointing” and “unacceptable”.

*The services are good except that we can’t access care in the weekends and at night times. During these times, we have to pay for drugs and other services.* (FGD6, 37 year old man, using ART)

*Woreda* health offices and some health centers noted that they lacked sufficient staff to efficiently and effectively perform routine administrative and clinical functions. High staff turnover was noted as a major challenge that is aggravated by inadequate incentives and staff retention strategies. Adding to the problem, support staff such as data clerks, case managers and adherence support workers was not paid their salaries on time. *Woreda* health offices staff said that they faced difficulties due to a lack of financial resources to mobilize the community to test for HIV and to provide care and support for needy PLHIV. They added that some *woreda* leaders have little regard for HIV prevention and control programs.

*I am the only person trained on ART and working in the unit. When I had to take time-off … there is no one who can work in this unit. Besides, trained staffs are frequently leaving the health facility.* (KII2, 26 year old man, HIV/AIDS program coordinator)

*We do not have budget even for routine tasks. … We don’t have budget for community mobilization and care and support for needy people. … There is an attitude problem with the leaders.* (KII14, 38 year old man, HIV/AIDS program coordinator)

### Policies and standards

#### Free access to care/Financing

Noting their impoverished living conditions and lack of food, PLHIV demanded free access to all medical care, not just ART drugs. Some said that when there are shortages of drugs to treat OIs, they struggled to pay for these drugs at ‘budget’ dispensaries (pay per dispensed drug) in the health facilities and at private drug stores. PLHIV were also asked to pay for laboratory services and inpatient care in hospitals regardless of their HIV status. However, these services were provided free of charge in the health centers. Hospital clients questioned why the procedures and financing policies were different at the two levels of public health care. Based on the national health policy, payment can be waived on condition the clients present a certificate from their respective *woreda* administration offices. Nevertheless, the shortage of drugs in public health facilities meant that they had no choice but to buy from private drug stores. When more than one family member was infected with HIV, medical expenses rose.

*Health centers are providing all health care free of charge. However, the care in the hospital is mostly payable. … We cannot afford buying them. Should we be punished for being poor?* (FGD2, 65 year old man, using ART)

*Now, we have to pay for everything at the hospital including inpatient care. … Families with many people with HIV are severely affected by this.* (FGD3, 30 year old woman, using ART)

Based on the experiences and expressed inability to afford to pay for medical care, PLHIV believed that “good services” such as good interaction with care providers and service setups were not sufficient for the health care to be acceptable.

#### Focus, implementation guidelines and service standards

According to health care providers and administrators, many health facilities had been constructed in the past five years in line with the country’s development plans and emphasis on expanding access to health care. Although the number of health facilities providing HATCS had increased, ART care was not available in many health centers. PLHIVs from remote areas that travelled 90 km or more, some of it on foot due to transport shortages and financial difficulties had limited access to HIV treatment.

*The country’s health policy is one of the best and delivering well. Accordingly, poor people are exempted. Due to expanding of health infrastructure, access to HIV care has increased. HIV treatment is provided free of charge although we had shortages of OIs drugs. Service delivery manuals and standards are very helpful and assist in quality service delivery.* (KII1, 32 year old man, care provider)

*Access to HATCS is very limited in our context. There are people coming from villages located four hours from here, often on foot, yet they [PLHIV] have to travel 90 km from here to get ART drugs.* (KII11, 28 year old man, head of health office)

PLHIV with financial difficulties were stressed due to fears of stopping HIV treatment. According to care providers and *woreda* officials, it was high time that ART centers were opened in those hard to reach areas. However, their efforts were not successful because of the large number of HIV positive people required and little regard for the remoteness of the *woredas*. PLHIV were thankful and happy about the national HIV/AIDS policy, acknowledging access to free ART and treatment of OIs.

*I come from very far place. I spend a lot from my limited income. …I have been struggling to survive. I become stressed when I fail to afford travel and food expenses. I don’t know if I can continue using HIV treatment unless the situations improve.* (FGD4, 32 year old man, using ART)

*Regarding opening new ART sites, priority has been given to woredas with larger number of HIV positive people. Due to this policy [directive], it has not been possible to open ART sites in distant places.* (KII19, 37 year old man, HIV/AIDS program coordinator)

*The HIV policy of our country is very good. It saved our lives. We thank the government for providing free ART and other services.* (FGD2, 45 year old man, using ART)

## Discussion

**Fig. 1:**
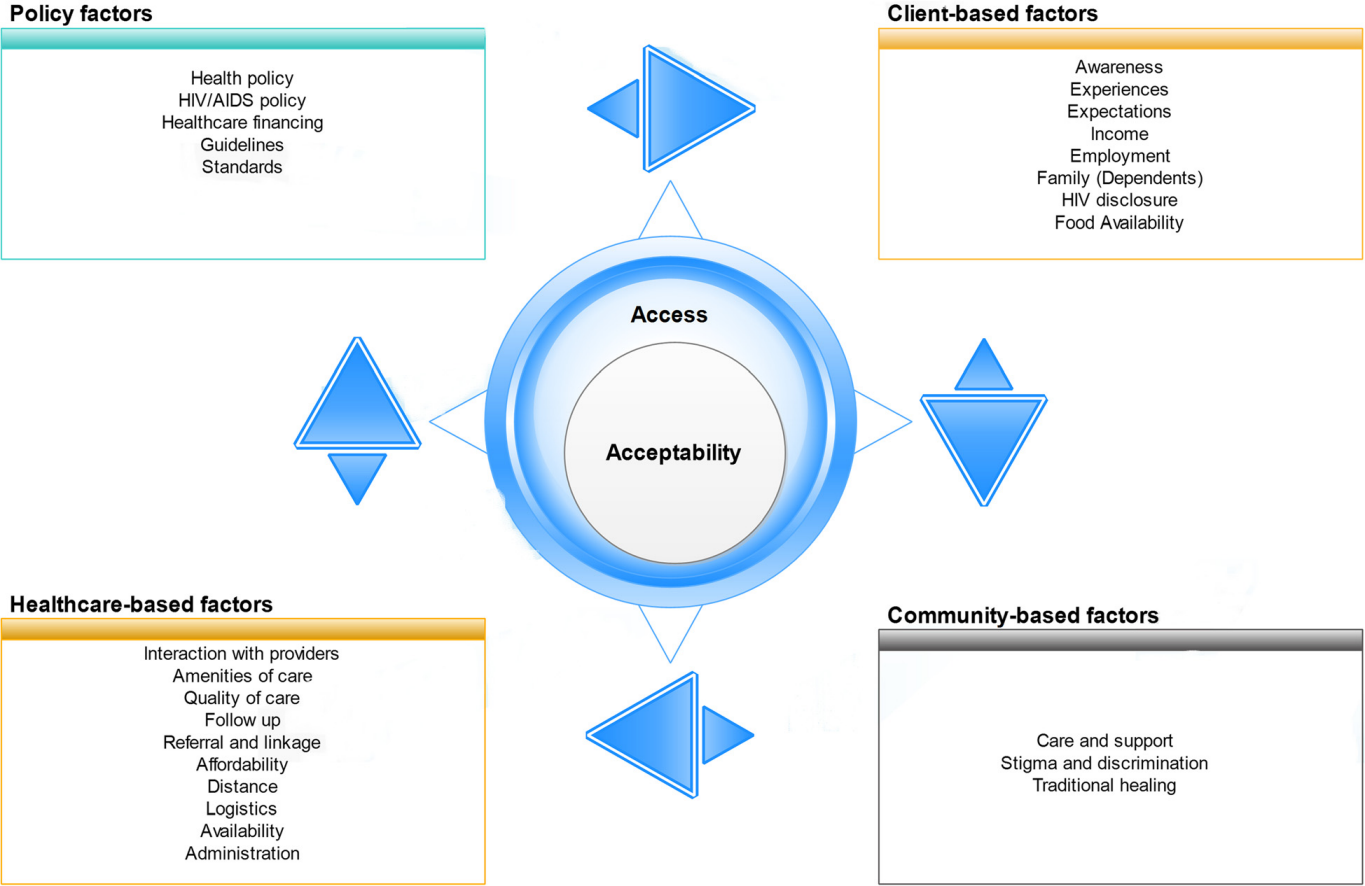
Socio-ecological factors of access to and acceptability of HATCS; Figure Legend: Acceptability is an element of access. They interact with and are impacted by four socio-ecological factors such as client-based, community-based, health system-based and policy factors

Much of the available literature on access to HIV care focuses on the geographical coverage (availability) of ART and the percentage of people currently on ART [29–31]. In addition, acceptability has been assessed in many studies in terms of willingness to enroll in or utilize health care, determined by clients’ backgrounds and preferences [32–34]. However, this study focused on determining the influence of the multiple factors that impact access to and accessibility of HATCS, including health care availability, responsiveness, affordability and financial fairness, quality and client preferences [11, 35–38]. In line with socio-ecological frameworks [16–18, 39] and propositions, the study’s findings are discussed under four units of analysis: client factors, community factors, institutional factors and policy and standards. As shown in Fig. 1, the discussion focuses on how each of these concepts was connected to each other and influenced access to and acceptability of HATCS.

### Client factors

The study revealed that client experiences and observations coupled with careful analysis of the risks and benefits influenced decisions about enrolling in HATCS. Positive and negative consequences perceived and/ or experienced amplified or limited the acceptability of HATCS, respectively. In addition, household characteristics such as food insecurity, unemployment and a lack of resources affected people’s perceptions of the value of life, choices made and intentions to attend health care. For instance, a lack of resources to care for children resulted in desperation and bitterness and failure to adhere to HATCS. Without basic resources, some PLHIV did not mind of stopping ART though they were aware of the consequences of doing so; this is consistent with the findings of a study in the northern part of Ethiopia [40]. Similarly, a study in the southwest of Ethiopia found that 63 % of those on ART suffered food insecurity and that this potentially impacted access and adherence to HATCS [41]. Given food insecurity and the presumed side effects of ART, standalone HATCS services may not succeed in ensuring access to HATCS [40, 42]. Interventions that aim to improve these services may need to integrate short-term food support programs and/ or create job opportunities to ensure self-reliance as a long term target.

Concealment of HIV status from spouses or sexual partners existed in an attempt to preserve relationships or prevent stigma and discrimination. Once again, this is consistent with the findings of other studies [43–45]. With the potential to result in late diagnosis and transmitting the virus to children, non-disclosure is an important public health problem that affects individuals’ access to treatment and care [42, 46]. In recognition of this fact, the Ethiopian policy on HIV/AIDS advises self-disclosure of HIV status to spouses that are given professional counseling. In the event that a person refuses to disclose to their partner, the policy acknowledges the right of the endangered partner to be informed of his/her partner’s HIV status so that he/she can access HIV care [47]. To what extent this has been implemented will remain a question to be answered by other studies. Targeted health education interventions on the importance of disclosing HIV status are essential to ensure that all those that need HATCS have access to it.

### Community factors

The findings show some reduction in stigma and discrimination against PLHIV and the increased role of family members, friends and the community in encouraging PLHIV to access HIV care. Nevertheless, stigma and discrimination still exist and undermine the acceptability of and adherence to HIV treatment, resulting in some PLHIV stopping treatment. Studies in Ethiopia have shown that HIV stigma and discrimination are due to misconceptions about transmission and the improper conduct associated with acquiring the virus [48–50]. Non-disclosure due to stigma was reported to result in maladaptive coping such as denial of HIV status, skipping doses, stopping ART or refusal to start HATCS [7, 51]. Increased health education and community engagement to tackle stigma and mobilization for care and support activities will be important, thereby enhancing access to and acceptability of HATCS.

The study found that the care and support (food, material and financial support) provided to needy PLHIV was inadequate, disorganized, short-lived and usually offered by individuals as a gesture of goodwill. Given food insecurity and financial difficulties, many PLHIV doubted the value of their life and either stopped or failed to adhere to HIV treatment. Studies in Ethiopia, Botswana and Cameroon have emphasized the need to include food and financial support to ensure access to, utilization of and adherence to HATCS [41, 42, 52, 53]. Care and support activities by family and community members were reported to boost self-worth that promotes positive coping such as using HATCS and adhering to prescriptions [54]. The findings suggest the need for well-coordinated and efficient resource mobilization to provide effective care and support for needy PLHIV.

Traditional healing emerged as an influential community-based resource that impacted utilization of HATCS. As previous studies in Ethiopia have shown, traditional medicine had wide reach and high acceptability compared to modern medicine [55]. In this study, it was reported that people had been cured of HIV after attending prayers, holy water and herbal medicine, were associated with ART stoppage or non-adherence. Similar findings were reported by studies in Ethiopia and other African countries [6, 8, 42, 56]. The findings suggest the importance of traditional medicine to access to and acceptability of HATCS. Training traditional healers and integrating their services into the formal healthcare system are important to promote access to HATCS. Furthermore, during the initiation and follow up sessions, care providers need to counsel PLHIV to adhere to prescriptions while using traditional healing services (if any).

### Institutional factors

Interactions with care providers were seen as very important aspects of care by PLHIV and affected their experiences with health facilities. A welcoming reception, good interactions and attention to their concerns resulted in contentment with HATCS. These characteristics show the importance of the ‘ways clients were treated’ that represented the *respect*, *communicatio*n and *attention* domains of health system responsiveness and were the *legitimate expectations* of clients [11]. Responsiveness has been reported as the determinant of satisfaction with healthcare and is associated with access to care [57, 58].

Disappointment with services, common with new providers, occurred when clients were disrespected and unheard, or their complaints were not attended to. HIV stigma in healthcare settings, such as avoiding physical examinations and unnecessary referral to other health facilities reduced acceptability of HATCS; this is consistent with the finding of another study in Ethiopia [59]. Therefore, improving health system responsiveness through training health care providers and avoiding stigma in healthcare settings may be required to enhance satisfaction with and acceptability of care in order to ensure access to care.

As health facilities faced a shortage of highly trained staff (general practitioners and specialist doctors), HATCS was provided by mid-level health professionals (health officers and nurses). This suggests that task-shifting has assisted in expanding access and maintaining HATCS without compromising quality and satisfaction with services. This has also been confirmed by other studies [60, 61]. However, the quality of care was affected when HATCS were provided by untrained staff, increasing the chances of malpractice and changes in treatment plans. Staff shortages and high staff turnover constrained service provision and impacted satisfaction with services. Health facilities need to ensure that service standards are adhered to in order to maintain the quality of care while the responsible authorities could apply incentives and staff retention strategies.

Service unavailability or inaccessibility, especially the treatment of OIs at night and on weekends and holidays challenged PLHIV who were unable to afford private care. Commensurate with available staff and budgets, health facilities could adopt strategies such as rotation-based staff assignment to avail access to care 24 h a day seven days a week. Shortages of HIV drugs, OIs drugs and laboratory reagents impacted service delivery and satisfaction with care. In extreme cases, they forced some PLHIV to stop ART. Due to the grave impact on access and acceptability, logistics and supply systems need to be efficient, effective and dependable to win clients’ trust and achieve the ambitious 90-90-90 targets of universal access to HATCS [62].

The study also found that all health facilities fell short in terms of ‘amenities of care,’ such as cleanliness, space, room arrangements, toilets, running water and facilities and access for people with disabilities. Maintaining ‘confidentiality’ and privacy was difficult as rooms are poorly arranged in some health facilities. Amenities of care and confidentiality are the *non-medical aspects of care* or *responsiveness* that impact clients’ experiences of health facilities [11, 63]. Studies show that client experiences of healthcare and responsiveness are associated with future intentions to access care and adherence to treatment [10, 11, 63]. Therefore, health facilities should work to improve clients’ experiences of services and improve responsiveness to increase access to and acceptability of HATCS.

### Policy and standards

The study showed that health centers and hospitals followed or implemented different financing approaches for HATCS. Inconsistencies in health financing, i.e., free vs paid OIs treatment respectively in health centers and hospitals, often resulted in dissatisfaction among paying clients as they felt discriminated against. The Ethiopian Health Policy as well as the Policy on HIV/AIDS stated that health care (including HATCS) should be on a pay per service basis with a waiver for those who could not afford to pay [47, 64], though ART services were later made free to all. Accordingly, those that could not afford to pay for treatment of OIs were exempt from payment. However, this was challenged by shortages, especially in hospitals and was cause for dissatisfaction with services. The Ethiopian Government has been piloting community-based health insurance schemes to avoid catastrophic health expenditure and increase access to health care [65]. Until such time as this is achieved, health facilities and local governments may be required to provide funding to procure essential logistics for the treatment of OIs and other medical conditions.

In an effort to fulfill policy promises and objectives, mainly to improve access, additional health facilities have been constructed in the zone and some facilities have been enlarged. Service delivery standards are useful tools to standardize and meet quality expectations. However, the difficulties encountered in opening new ART sites in remote *woredas* require attention and should be solved in conformity to the national ART implementation guideline [66] to improve access to HATCS with less catastrophic expenditure. Therefore, the authorities may need to reevaluate the situation in disadvantaged and remote areas.

### Strengths and limitations of the study

The strength of the study was this was a novel qualitative study that collected data from multiple sources to understand the contexts and factors involved in access to and acceptability of HATCS. Data analysis was rigorous and was assisted by software. As a limitation, due to the nature of the study, subjectivity during data analysis and interpretation could not be totally avoided, although validation of the preliminary findings was conducted with experts. The findings of the study apply to Wolaita Zone; however, they can be considered reference material to initiate studies and projects in similar contexts.

## Conclusions

The study demonstrated that a socio-ecological perspective offers a useful framework to evaluate the interplay among the complex and multilevel factors that impact access to and acceptability of HATCS. The findings of the study are summarized as follows. For a better understanding of access to and acceptability of HATCS, the socio-ecological perspective provides four interactive levels such as clients, community, health system and policy and standards (macro factors). Programs and interventions planning to improve access may use this framework.

The study findings have the following implications for practice and research. . Health systems need to train staff on client-provider interactions in order to improve health system responsiveness and avoid stigma in healthcare settings, thus improving healthcare experiences. PLHIV require a training on treatment adherence, the importance of HIV disclosure and coping skills to do away with stigma and discrimination. Similarly, health systems need to improve their logistics supply system, and adopt strategies to motivate and retain staff and avail services at all times. Continued community education on HIV/AIDS may be required to dispel HIV stigma and discrimination, and to enhance community participation in care and support activities. As the acceptability of and access to HATCS was linked to food availability and financial resources, it will be important to incorporate short and long term plans (food assistance for acute problems and seed money to open small enterprises) may be essential for needy PLHIV. In addition, those responsible to open new ART sites should follow the implementation manual while care providers should continue to use service delivery standards to maintain the quality of care. Further studies will be required to confirm the study findings.

## Additional file

Additional file 1:
**Study Instruments.** (DOCX 16 kb)

## Abbreviations

ART: Antiretroviral therapy
FGD: Focus group discussion
HATCS: HIV/AIDS treatment and care services
IDIs: Individual in-depth interviews
KIIs: Key informant interviews
PI: Principal investigator
PLHIV: People infected with HIV
RA: Research assistant
WHO: World Health Organization
