# Supplementary material for: A socio-ecological perspective of access to and acceptability of HIV/AIDS treatment and care services: a qualitative case study research
Source: BMC Public Health. 2016 Feb 16;16:155. doi: 10.1186/s12889-016-2830-6 (PMC4754879; doi:10.1186/s12889-016-2830-6)
Supplement: Additional file 1: — Study Instruments. (DOCX 16 kb) [file 12889_2016_2830_MOESM1_ESM.docx]

**Supplementary document 1**

**Interviews and FGDs guides**

The following are summary of key guiding questions prepared for each group of participants selected for the study. Each of the questions were followed by probes and follow up questions as needed during the inquiry.

1. Instrument for KIIs for health administrators, service providers, coordinators of the associations of PIWH, case managers, adherence support workers, health extension workers and community volunteers:

*How do you see the healthcare services provided to people living with HIV?*

*How acceptable are HATCS to you and to PIWH?*

*How accessible are HATCS?*

*How are HATCS financed? How do you see the role of traditional healing services in HATCS?*

*What are the challenges related to providing HATCS? What should be improved and how?*

*In addition, for service providers, how convenient your service is to your clients? How satisfied are they? How are your interactions with the clients?*

1. Instrument for IDIs with traditional healers:

*What is your opinion about how and why people become sick?*

*What services do you provide to your clients and to PIWH?*

*After providing care, what do you advise your clients ‘to do’ or ‘not to do?’ Why?*

*How effective and acceptable your services are to your clients?*

*How are your relationships and collaboration with health facilities?*

*Do you make referrals? Where and why?*

*What are the challenges and what do you recommend for the future?*

1. Instrument for IDIs with persons stopped ART:

*What are your experiences of living with HIV?*

*How are you taking care of your health?*

*What made you stop ART?*

*What do you feel about HIV care in health facilities?*

*What are/were the challenges you face/d that affected the use of HIV care services?*

*How acceptable were/are HATCS in the health facilities?*

*What are your intentions about ART use in the future?*

*What would motivate you restart ART?*

*What changes should be made regarding HIV care?*

1. Instrument for FGD with people using HIV /AIDS treatment and care services

*How does it feel living with HIV and how are PLHIV treated in your community?*

*What do you feel about HIV care services in health facilities?*

*How is access to HIV care services for you?*

*What do you feel about the interactions with the care providers?*

*How do you see the role of traditional healing services on HIV care services?*

*How are HIV care services financed in the health facilities?*

*What challenges do you face in accessing HIV care services?*

*How acceptable HIV care services are for you?*

*What should be improved in the future?*

1. Instrument for FGD with general community members

*How are people infected with HIV treated in your community?*

*What are your experiences of social life with people infected with HIV?*

*What are the roles of traditional medicine in HIV care?*

*What HIV treatment and care services are available in your community?*

*Where do people go when they need HIV care?*

*How acceptable and accessible are these services?*

*What are the challenges of accessing and using HIV care?*

*What changes should be made or improved?*
